# Mutual coupling and synchronization of optically coupled quantum-dot micropillar lasers at ultra-low light levels

**DOI:** 10.1038/s41467-019-09559-2

**Published:** 2019-04-04

**Authors:** Sören Kreinberg, Xavier Porte, David Schicke, Benjamin Lingnau, Christian Schneider, Sven Höfling, Ido Kanter, Kathy Lüdge, Stephan Reitzenstein

**Affiliations:** 1 Institut für Festkörperphysik, Technische Universität Berlin, Hardenbergstraße 36, 10623 Berlin, Germany; 2 Institut für Theoretische Physik, Technische Universität Berlin, Hardenbergstraße 36, 10623 Berlin, Germany; 3 Technische Physik, Universität Würzburg, Am Hubland, 97074 Würzburg, Germany; 4 SUPA, School of Physics and Astronomy, University of St. Andrews, St. Andrews, KY16 9SS UK; 5 Gonda Brain Research Center and Department of Physics, Bar-Ilan University, Ramat-Gan, 52900 Israel

## Abstract

Synchronization of coupled oscillators at the transition between classical physics and quantum physics has become an emerging research topic at the crossroads of nonlinear dynamics and nanophotonics. We study this unexplored field by using quantum dot microlasers as optical oscillators. Operating in the regime of cavity quantum electrodynamics (cQED) with an intracavity photon number on the order of 10 and output powers in the 100 nW range, these devices have high *β*-factors associated with enhanced spontaneous emission noise. We identify synchronization of mutually coupled microlasers via frequency locking associated with a sub-gigahertz locking range. A theoretical analysis of the coupling behavior reveals striking differences from optical synchronization in the classical domain with negligible spontaneous emission noise. Beyond that, additional self-feedback leads to zero-lag synchronization of coupled microlasers at ultra-low light levels. Our work has high potential to pave the way for future experiments in the quantum regime of synchronization.

## Abstract (web summary)

The synchronisation of optical cavities in the quantum regime is still relatively unexplored. Here, Kreinberg et al. investigate the coupling behaviour of two quantum dot microlasers and find that spontaneous emission noise from cavity QED effects plays an important role.

## Introduction

Synchronization is an ubiquitous phenomenon in mutually coupled systems^1^ which—under appropriate conditions—leads to a spontaneous self-organization of the coupled elements^2^. A multitude of different physical, biological, or chemical systems can exhibit synchronization, making it a fundamental interdisciplinary property of interacting nonlinear systems^1,3,4^. The complexity of this phenomenon is well depicted by the variety of existing synchronization scenarios. One prominent example is chaos synchronization, where the individual coupled elements all follow the same chaotic trajectory^5^. In this context, semiconductor lasers are attractive table-top devices to study fundamental aspects of nonlinear dynamics and synchronization^6–15^ with proposed applications in random number generation and secure key exchange^16,17^.

Recently, the prospect of exploring synchronization in coupled nanoscale oscillators has received increasing attention. Enabled by important technological advances, it has become feasible to investigate nonlinear dynamics and synchronization at ultra-low energies in systems previously only explored from a quantum mechanical perspective. For instance, mutual synchronization of the Kuramoto type has been demonstrated in optomechanical structures^18^ and in nanomechanical oscillators^19,20^. Most interesting is the quantum limit of nonlinear interaction and synchronization, which has been addressed experimentally for instance in 2D Josephson junction arrays^21^. It has also triggered numerous theoretical studies which predict novel phenomena such as partial locking and synchronization blockade^22,23^, even elucidating interesting connections between entanglement and synchronization^24–26^.

Situated at the crossroads between nonlinear dynamics, nanophotonics and quantum optics, cavity-enhanced microlasers are interesting devices to drive research on synchronized oscillators toward the quantum regime. They offer a rich spectrum of exciting physics with potential applications as coherent light sources in system-on-chip quantum technologies^27^. Due to their low-mode volume on the order of the cubic wavelength, microlasers usually operate in the regime where cavity quantum electrodynamics (cQED) effects such as enhanced spontaneous coupling in terms of high (*β*) factors become important. Up until now, microlaser studies have focused almost exclusively on the properties of individual devices, not considering coupling interactions with external passive or active elements. However, this situation is changing and recent works report on interesting effects like spontaneous symmetry-breaking due to local coupling between cavity modes in nanophotonic structures^28,29^ and on tailoring of the mode-switching dynamics and photon statistics in feedback coupled microlasers^30,31^. At the same time it has become interesting to theoretically describe the dynamics and stability of microlasers when mutually coupled with delay^32–34^. Beyond that, if it comes to scaling effects and the pursuit for better understanding of coupling and synchronization in complex small-scale systems in the presence of enhanced noise, our work may also foster progress in related disciplines like socioeconomics, biology, ecology, and hydrodynamics^35–37^.

Here, we present the experimental implementation of optically coupled microlasers with incoherent optical coupling delay at far-below µW output power levels. We apply bimodal semiconductor quantum dot (QD) micropillar lasers driven with intracavity photon numbers on the order of ten to study mutual coupling at ultralow light levels. This detailed investigation on the dynamics of coupled micro- or nanolasers is of interdisciplinary and immediate importance for scientists working on the dynamics of nonlinear oscillators and for those interested in microscopic or nanophotonic lasers. In the studied devices the energy degeneracy of the fundamental cavity mode is lifted by slight structural asymmetries resulting in two orthogonal linearly polarized fundamental mode components^38–40^. The related bimodal behavior is of essential importance for the dynamic properties of micropillar lasers and leads to a plethora of exciting physical phenomena such as gain competition^41^, unconventional normal-mode coupling^42^, and mode switching^43^ as an instance of Bose–Einstein condensation of photons^44^. Using bimodal QD-microlasers we experimentally demonstrate mutual locking and synchronization via a detailed analysis of their spectral properties and photon statistics in face-to-face configuration and in presence of an additional passive delay. Accurate numerical modeling supports our findings and allows us to reveal the underlying time-resolved character of the synchronized dynamics.

## Results

### Emission characteristics of bimodal micropillars

**Fig. 1:**
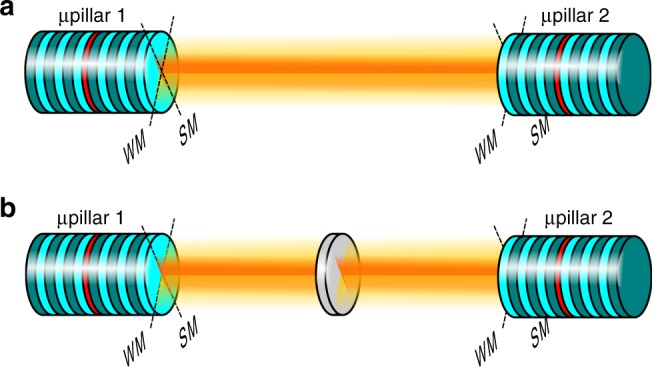
Illustration of the studied experimental configurations. **a** Face-to-face mutual coupling and **b** mutual coupling via passive relay of two micropillar (μpillar) lasers. The setup is arranged to couple the two perpendicularly polarized emission modes of each micropillar (i.e. strong mode (SM) and weak mode (WM)) to their respective counterparts

The QD microlasers, which we used for coupling experiments as sketched in Fig. 1, were realized by means of molecular beam epitaxy of planar microcavity structures and subsequent nanoprocessing of electrically driven micropillar cavities as we detail in Supplementary Note 1. These devices are delicate objects at the forefront of science and technology and their optical properties, for instance in terms of the emission energy, vary within certain bounds from microlaser to microlaser. While this variation is not critical for fundamental studies of individual microlasers as done in many previous works, it becomes a major issue in case of coupling scenarios between different microlasers with an effective injection locking range on the order of a few gigahertz^45^. In our present work the situation is even more problematic since we do not only require spectral matching of the microlasers, they also need to show very similar emission properties with respect to their output power and linewidth to enable symmetric mutual coupling and synchronization experiments. For this purpose we fabricated large linear arrays each consisting of 120 QD-microlasers from which we selected pairs of suitable candidates as described in Supplementary Note 1 in more detail.

**Fig. 2:**
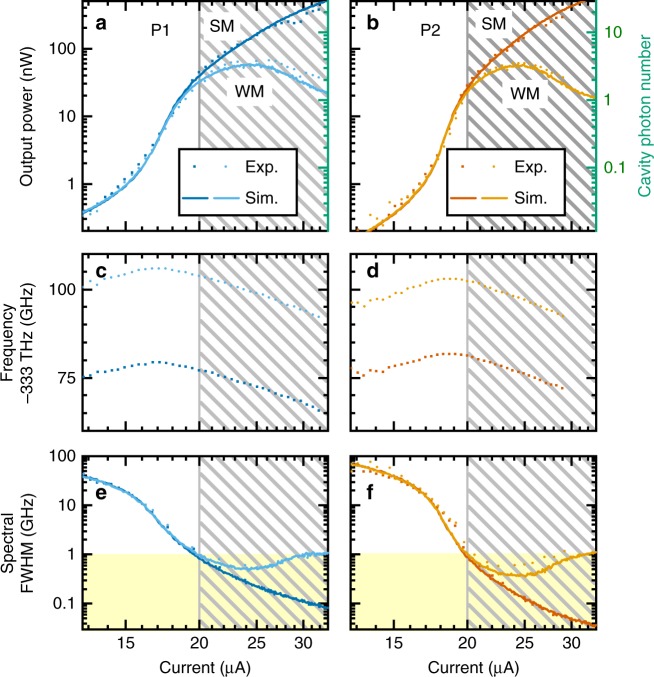
Input–output characteristics of two QD-micropillar lasers used in mutual coupling experiments. Experimental (dots) and numerical (lines) injection current dependence of output power, emission frequency and linewidth for the strong modes (SM) and weak modes (WM) of pillar P1 (panels **a**, **c**, and **e**) and pillar P2 (panels **b**, **d**, and **f**). Dashed areas indicate the operation regime for the mutual coupling experiments which require emission linewidths smaller than one gigahertz (yellow area) and can be achieved for injection currents exceeding 20 μA. The calculated intracavity photon number presented in panels **a** and **b** ranges from about 1 and 20 in the relevant current regime and was determined numerically by our theory

Injection current emission characteristics of a particular suitable pair of microlasers with almost identical emission properties are depicted in Fig. 2. The experiments were performed using the spectroscopic setup described in the Methods section and in Supplementary Note 1 in more detail. The strong mode (SM) of each of the two lasers, which are used in mutual coupling experiments, shows a characteristic s-shape in log–log presentation of the input–output curves and the slight nonlinearity indicates a high *β*-factor. The weak mode (WM) of each laser loses the intermodal gain competition^43^, saturates at intermediate injection current and decreases in intensity at highest pump conditions. The description of the experimental data by our theoretical model (see Methods) yields experimentally not accessible parameters (summarized in Table 1) such as the spontaneous emission factor of *β* ≈ 4 × 10^−^^3^ for both microlasers and the injection current dependent intracavity photon number. As can be seen in Fig. 2a, b this photon number is as low as 1–20 in the working range (shaded areas) of our coupling experiments. The SM and WM emission frequencies plotted in the middle panels of Fig. 2 shift to higher values with increasing pump due to the plasma effect until a red shift sets in at high injection currents because of sample heating. The frequency splitting between SM and WM is 26 GHz for pillar 1 and 21 GHz for pillar 2 and stays constant over the investigated pump current range (see Methods for high resolution spectra and more information). As presented in the lower panels of Fig. 2 the SM linewidths decrease by more than two orders of magnitude eventually narrowing down to less than 100 MHz at the highest injection currents of about 30 μA. In contrast, the WM’s above-threshold linewidths increase, which indicates a noncomplete transition to laser action for these modes. Noteworthy, previous injection-locking experiments on QD-micropillar lasers revealed a locking range of approximately 1 GHz^45^. Thus, to resolve possible locking effects between two mutually coupled micropillar lasers, emission linewidths smaller than the expected locking range of approximately 1 GHz are required (yellow areas in Fig. 2) which defines the working range (shaded areas in Fig. 2) of the microlasers for the coupling experiments discussed in the following.

**Table 1:** Parameters used for the simulations if not stated otherwise

Fitted parameter	Value
Optical cavity losses, strong (weak) mode	*κ*_s_ (*κ*_w_) 39 (38.5) ns^−1^
Optical gain coefficient, strong (weak) mode	$g_{\mathrm{s}}^0$ ($g_{\mathrm{w}}^0$) 5.35 (5.21) $\frac{{{m}^2}}{{{V}^2{s}}}$
Self gain compression, strong (weak) mode	*ε*_ss_ (*ε*_ww_) $10 (12) \times 10^{ - 10}\frac{{m^2}}{W}$
Cross gain compression, strong (weak) mode	*ε*_sw_ (*ε*_ws_) $16 (17.8) \times 10^{ - 10}\frac{{m^2}}{W}$
Spontaneous emission factor	*β*^P1(P2)^ 3.5 (4) × 10^−3^
Parasitic current	$J_{\mathrm{p}}^{{\mathrm{P1}}({\mathrm{P2}})}$ 2.3 (7.3) μA
Injection efficiency	*η*^P1(P2)^ 0.0596 (0.0674)
Linewidth enhancement factor	*α*^P1(P2)^ 1.7 (1.0)
Reservoir carrier lifetime	*τ*_r_ 1 ns
Given parameter	Value
Effective scattering rate	*S*^in^ 7 × 10^−15^ m^2^ ps^−1^
Effective lasing mode area	*A* 15 µm^2^
Lasing mode volume	*V* 5 µm^3^
Number of (in)active QDs	$Z_{{\mathrm{inact}}}^{{\mathrm{QD}}}$ 312 (938)
Background refractive index	*n*_*bg*_ 3.34
QD lifetime, μ-laser 1 (2)	$\tau _{{\mathrm{sp}}}^{{\mathrm{P1}}({\mathrm{P2}})}$ 155 (185) ps
Photon energy	ℏ*ω* 1.38 eV
Coupling delay time	*τ* 3.85 ns

### Frequency locking of mutually coupled microlasers

**Fig. 3:**
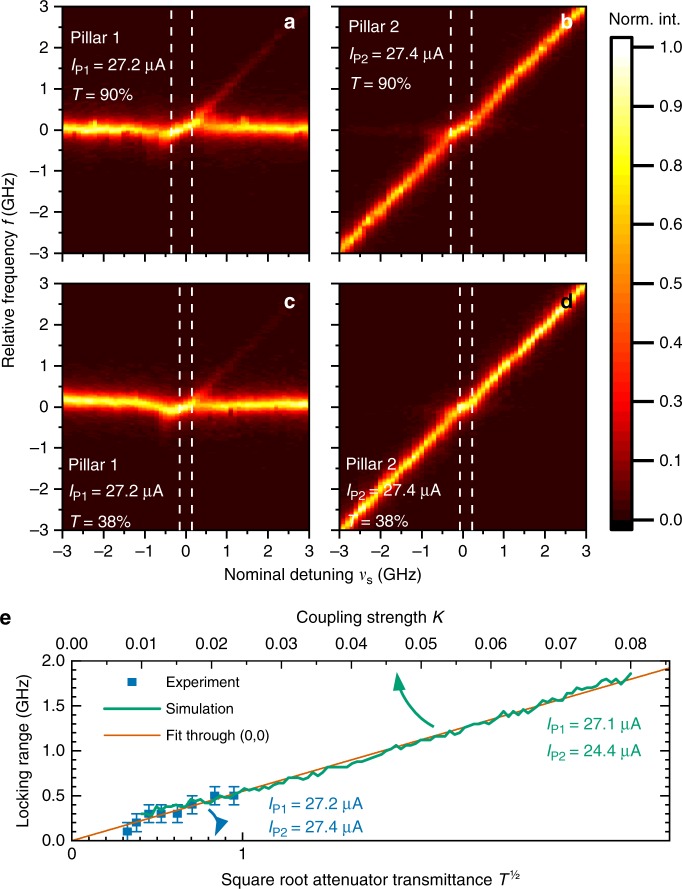
Mutually coupled strong modes of the two micropillar lasers for different coupling strengths. **a** Detuning scans of the strong modes with high (panels **a** and **b**) and low (panels **c** and **d**) coupling strengths *K*. *T* is the transmittance of the attenuator in the coupling path. **e** Dependence of the locking range on the square root of the attenuator transmission (lower axis) and the coupling strength *K* used in numerical simulations (upper axis). The horizontal axes are scaled such that experimental and simulated data both lie on a common linear function

We first study the spectral properties of our coupled optical oscillators in face-to-face configuration, unveiling a coherence behavior and locking properties particular to high-*β* microlasers. Therefore, we mutually couple the selected pair of micropillar lasers and vary the relative detuning between the two microlasers as shown in Fig. 3a–d. The emission frequency of pillar 1 (P1) is kept constant (at constant temperature of 32 K). Meanwhile the frequency of pillar 2 (P2) is precisely scanned across the emission frequency of pillar 1 by sweeping its temperature in the range *T*_2_ ∈ [32, 36 K]. While the temperature is swept, emission spectra of pillar 1 are recorded by using a Fabry–Perot scanning interferometer. A matrix is formed from the spectra, such that each column of the matrix corresponds to one spectrum. Emission spectra of pillar 2 are recorded in the same way in a second run. The matrices are then plotted as 2D heat maps. The detuning ranges of ±3 GHz displayed in Fig. 3a–d correspond to a temperature scan ranging from 34.9 to 33.9 K. When tuning the two lasers close to resonance, i.e., for detunings $\lesssim 0.5\,{\mathrm{GHz}}$, clear mutual frequency locking can be identified as a change in slope of the relative frequency vs. detuning characteristics: within the locking range, the emission of both lasers is shifted toward a common frequency, returning to their free-running values outside of the locking range. A comparison between panels a–d of Fig. 3 illustrates that the locking range depends on the mutual coupling strength (varied by adjusting the variable attenuator in the coupling path), which has a transmittance *T* of 90% (38%) in a–d.

Deeper insight into the locking behavior requires a more detailed study of the locking range as a function of the coupling strengths. In agreement with previous reports on externally controlled micropillar lasers^45^ and coupled semiconductor lasers^33,46^, the obtained locking-range width is proportional to the injected electric field strength, i.e., to the square root of light intensity controlled by the attenuator transmittance (*T*). Figure 3e depicts such dependence for both experimental (symbols) and simulations (solid line) data. In order to plot both datasets together, we match the linear dependence of the locking range on *K* (numerics) with the experimental data to find the proportionality factor between *K* and the square root attenuator transmittance. Even though measuring the free space optical losses (beam splitters, polarization optics, lenses and cryostat windows) is in principle feasible, this matching is necessary because it is not possible to quantify the coupling efficiency into the pillar and to the laser field. The maximum experimental amplitude coupling strength (at *T* = 1) is thus estimated as *K* ≈ 2.5%. In the simulations (solid lines), the coupling strength *K* is studied over a larger range.

### Identification and analysis of mutual coupling

**Fig. 4:**
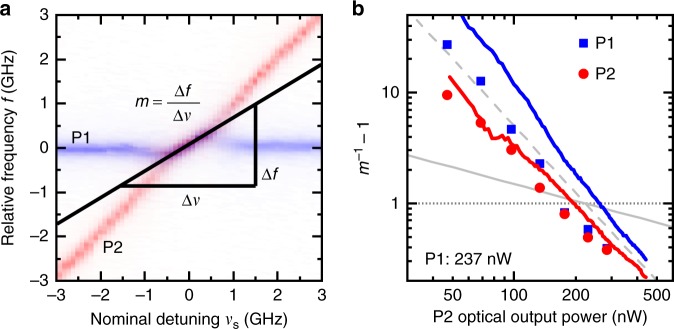
Locking slopes of the two mutually coupled micropillar lasers. Panel **a** illustrates how the slope *m* is calculated. **b** Experimental (symbols) and numerically simulated (lines) locking slopes in dependence of the optical output power of pillar P2. The horizontal dotted line depicts the classically expected slope *m* = 0.5 and the oblique dashed and continuous gray lines respectively correspond to the slopes of *A* = −2 and *A* = −0.5 (Please see the main text for the definition of *A*)

The presence of locking between the microlasers emission unequivocally indicates coupling. However, it is the slope *m* described by the microlasers’ emissions inside the locking region (see Fig. 3a–d), which determines the direction of the coupling. Figure 4a depicts this slope as the ratio of frequency change of the locked signal Δ*f* and the nominal detuning Δ*ν* between the laser modes. We use this slope as the indicator for having achieved not only unidirectional but mutual coupling. Consider for instance the limiting case of a unidirectional injection experiment: here the emission of the injecting master laser by definition must not be influenced by the slave laser subjected to injection. Strictly speaking this condition can only be fulfilled by placing optical isolators in the coupling path. However, provided that the output powers of the two mutually coupled lasers are at least strongly imbalanced, there will be one “master-like” laser and one “slave-like” laser, even without an optical isolator. While the former is almost unaffected by the mutual coupling, the latter is strongly influenced by the injected light. In this situation, when tuning the master-like laser, the slave-like laser will perfectly follow the injected signal in the locking region, which results in a locking slope of *m* = 1. On the contrary, if only the slave-like laser is tuned, the locking slope will have a value of 0, because its emission frequency is locked to the master-like laser. If the output power imbalance between master and slave is reduced, the locking slope will start turning away from these extreme values and eventually reach *m* = 0.5 for evenly balanced coupling (cf. horizontal dotted line in Fig. 4b).

Based on these considerations, for phase-locked (or frequency-locked) lasers under mutual coupling conditions, the locking slopes of both oscillators, *m*_P1_ and *m*_P2_, should be equal, as both lasers are locked to each other and emit light on a frequency in between the two free-running laser lines. Surprisingly, the two microlasers exhibit different locking slopes, both in experiment and simulations. In Fig. 4b, the inverse slopes $m_{{\mathrm{P1}}}^{ - 1}$ and $m_{{\mathrm{P2}}}^{ - 1}$ can be seen to differ especially for low-output powers of pillar P2, which resembles a master–slave setup for which *m*_P2_ = *m*_P1_ = 0 is expected. This means that inside the locking range, the average emission frequency of the two microlasers is deviating proportionally to the nominal detuning. Importantly, these deviations are not expected to occur for classical coupled oscillators and are attributed to the effect of partial locking in high-*β* microlasers^45,47^. The fact, that the locking slopes get more similar when the output power of pillar P2 is increased, can be explained by two converging factors: (a) the stronger injection into pillar P1 and (b) the decreasing relative contribution of quantum noise to the output power of pillar P2.

The experimental and numerical observations in Fig. 4b are further analyzed reducing our laser model to a system of coupled phase oscillators as detailed in the Methods section. Based on Eq. (10) we conclude that for fixed output power of pillar P1 this description yields that *m*_P2_ depends on the output power *P*_out,2_ of pillar P2 via

1 $$(P_{{\mathrm{out,2}}})^A \propto m_{{\mathrm{P2}}}^{ - 1} - 1,$$

with a scaling factor $A = - \frac{1}{2}$. Thus, theory predicts that the common frequency within the locking range gets pulled closer toward the free-running frequency of P2, i.e., $m_{{\mathrm{P2}}}^{ - 1} - 1 \to 0$, with increasing power of pillar P2. The experimental scaling coefficient *A*_exp_ is obtained by fitting the equation

2 $$B \cdot (P_{{\mathrm{out,2}}})^{A_{{\mathrm{exp}}}} = m_{{\mathrm{P2}}}^{ - 1} - 1{,}$$

to the experimental data. In contrast to the analytic expectations, the experimental data and numerical simulations suggest an exponent of *A* ≈ −2 instead of the expected $A = - {\textstyle{1 \over 2}}$ (see respective dashed and gray continuous lines in Fig. 4b. The different A coefficients of −1/2 and −2 are explained by the fact that the solution of the simplified coupled phase equations in Eq. (2) describes the behaviour of individual Fabry–Perot fine structure modes (c.f. Supplementary Note 2) that are not resolved in experiment. However, in the experiment and in our numerical QD laser model (Eqs. (4)–(6)), all coupled-cavity modes contribute and, after fitting a Lorentzian profile to the envelopes, both results agree very well and lead the slopes of −2 in Fig. 4. We refer to Supplementary Note 3 for more information on the numerical simulations of the scaling coefficient. In future experiments, it will be interesting to study the locking slopes with higher spectral resolution (of better than 10 MHz) to access the regime predicted by the solution of the coupled phase equations.

### Identification of synchronization by correlation studies

In the field of cavity-enhanced nano- and microlasers a detailed study of the photon statistics of emission is of particular interest. Measuring the power-dependent photon autocorrelation function *g*^(2)^(*τ*) allows for instance to unambiguously prove lasing emission in high-*β* lasers operating close to the limit of thresholdless operation^48^, for the identification of superradiant emission^49^, or for ruling out chaotic mode switching^45^. In addition, it is also highly beneficial for the identification of chaotic dynamics in feedback coupled microlasers operating at ultra low-emission powers^30^.

Determining the photon auto- and cross-correlation function is also highly interesting in the present case of mutually coupled microlasers to obtain profound insight into the underlying emission dynamics and possible synchronization of intensity fluctuations. Due to the intrinsic mode competition in micropillar lasers, we expect mode-switching events during which the SM is dark and the WM is bright. The frequency of these mode-switching events and the related intensity variance are enhanced in the present case of high-*β* lasing^43^.

**Fig. 5:**
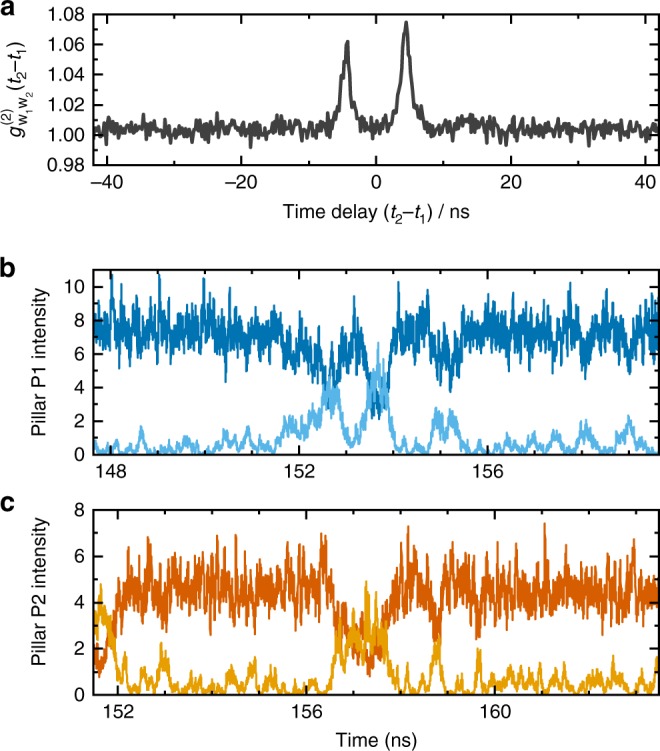
Intensity cross-correlations of two coupled micropillar lasers. **a** Intensity cross-correlation $g_{w_1w_2}^{(2)}(t_2 - t_1)$ of the weak modes of pillars P1 and P2. The weak modes were tuned to resonance (*ν*_w_ = 0) in face-to-face configuration (see configuration a in Fig. 1) at injection currents *I*_P1_ = 27.7 μA and *I*_P2_ = 24.5 μA. The two main peaks at 4.3 ns suggest leader-laggard synchronization of the intensity fluctuations between the lasers. One roundtrip (7.7 ns) further, at 12 ns, weaker revival peaks are barely observable. **b, c** Simulated intensity dynamics, showing the leader-laggard behavior of the two coupled micropillars P1 (panel **b**, upper trace: strong mode, lower trace weak mode) and P2 (panel **c**, upper trace: strong mode, lower trace weak mode) at injection currents of *I*_P1_ = 27.1 μA and *I*_P2_ = 24.2 μA. The time axis for pillar P2 has been shifted with respect to pillar 1 by 3.85 ns, i.e., the optical distance between the two micropillars. This illustrates the delayed correlation of the intensity fluctuations

In the respective experiment the output intensities of pillar P1 and pillar P2 are cross correlated via single-photon counting module (SPCM) 1 and SPCM 2 as presented in the Methods section and more detailed in Supplementary Figure 1, respectively. Polarization optics are used to flexible detect photons from any polarization mode of pillars P1 and P2. We focus in our study on the case where the WMs are resonantly coupled and feature pronounced intensity fluctuations, as the SMs show only marginal signatures of photon bunching and no significant cross-correlation peaks when resonantly coupled. As reference we present auto-correlation functions of noncoupled pillars P1 and P2 in Supplementary Figure 7 and briefly discuss the observations in Supplementary Note 5. Noteworthy, pronounced photon bunching in auto- and cross-correlation functions is a typical behavior in delay-coupled micropillar lasers^30^. We denote the second-order photon correlation function of the WMs as $g_{{\mathrm{w}}_i{\mathrm{w}}_j}^{(2)}$, giving the auto-correlation for pillar *i* when *i* = *j*, and the cross-correlation for *i* ≠ *j*. An example of a WM–WM cross-correlation measurement is shown in Fig. 5a for pump currents of *I*_P1_ = 27.7 μA and *I*_P2_ = 24.5 μA. Clear peaks can be observed at *t*_2_ − *t*_1_ ≈ 4 ns, corresponding to the coupling delay of 3.85 ns between the microlasers. The double-peak structure indicates leader-laggard intensity synchronization of the two micropillars, i.e., if a fluctuation occurs in pillar P1, there is a chance that it will be repeated in pillar P2 and vice versa. The numerical time series depicted in Fig. 5b confirm this interpretation of the experimental data in terms of leader-laggard dynamics^32^, showing a strong similarity between the time-series when either of the time-series is shifted in time by the coupling delay *τ*. The laser coupling can be observed to irregularly induce short mode-switching events in both lasers (e.g., near *t* = 153 ns for pillar P1 in Fig. 5b). The relatively low-peak values of the cross-correlation $g_{{\mathrm{w}}_1{\mathrm{w}}_2}^{(2)}(\tau )$ in comparison to the free-running auto-correlation ($g_{{\mathrm{w}}_1{\mathrm{w}}_1}^{(2)}(0) = 1.5$ for pillar 1 and $g_{{\mathrm{w}}_2{\mathrm{w}}_2}^{(2)}(0) = 1.6$ for pillar 2) proves imperfect synchronization between the lasers, and suggests that only a small ratio (≈13%) of switching events are repeated in the respective other laser.

**Fig. 6:**
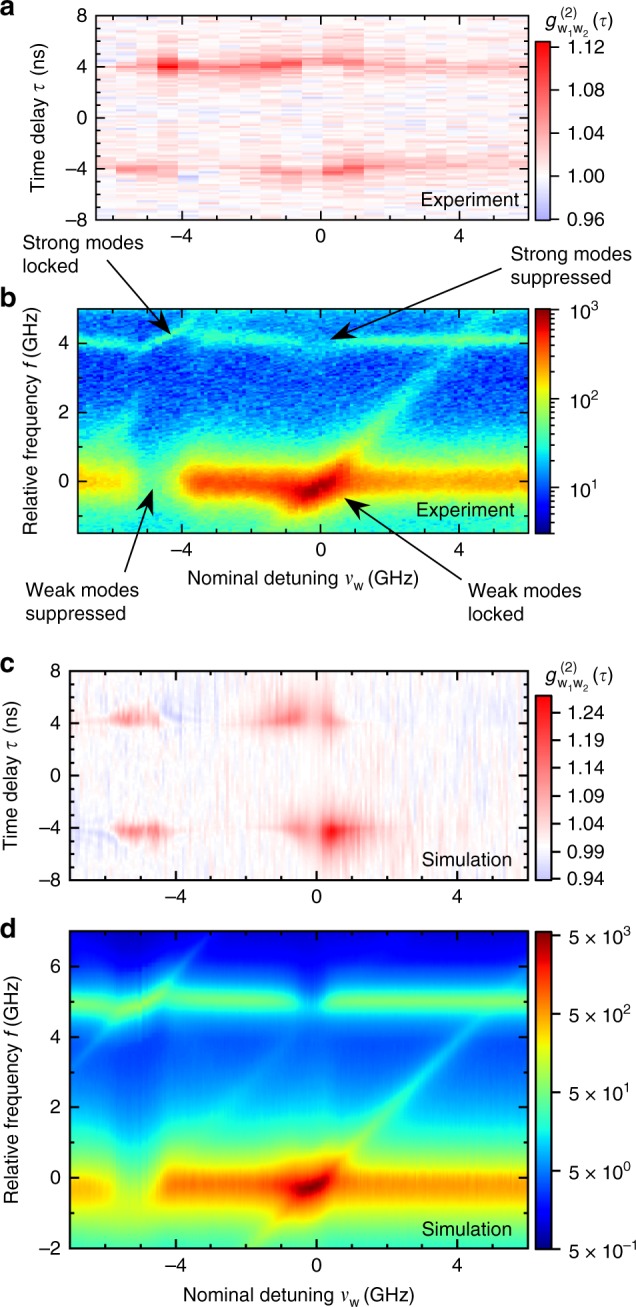
Correlation and locking maps of two coupled micropillar lasers. Measured **a** and simulated **c** weak-mode intensity cross-correlation $g_{{\mathrm{w}}_1{\mathrm{w}}_2}^{(2)}(\tau = \tau _2 - \tau _1)$ (color-coded) in face-to-face configuration as function of the time delay *τ* for different detunings *ν*_w_ (*I*_P1_ = 28.0 μA and *I*_P2_ = 25.6 μA). **b**, **d** Corresponding log-intensity Fabry–Perot interferometer (FPI) spectra (color-coded) of the laser output in dependence of the detuning *ν*_w_. *I*_P1_ = 28.6 μA, *I*_P2_ = 27.0 μA in the experiments, *I*_P1_ = 27.1 μA, *I*_P2_ = 24.4 μA in the simulations

The intensity cross-correlation depends on the dynamical susceptibility of the lasers to a perturbing signal, and thus on their ability to reproduce and synchronize to the signal of the other laser. We, therefore, investigate the dependence of the cross-correlation on the mutual laser detuning *ν*_w_ of the WMs. Figure 6a, b shows the measured cross-correlation of the WMs of the two lasers and the FPI spectra of the WM, respectively. Since the SM is much more intense than the WM, it is still visible in the FPI spectra on a logarithmic scale even after attenuation by the polarizing beam splitter (PBS). Interestingly, as we discuss in Supplementary Note 6, mutual optical coupling does not significantly influence the overall intensity in detuning dependent locking experiments. The mutual locking of the WMs around a WM detuning of 0 leads to a strong enhancement of the WM signals, while suppressing the SM intensity. Near the locking range of the SMs, at a WM detuning of *ν*_w_ ≈ −5 GHz, the reverse effect is observed together with a strong suppression of the WMs. This can be understood by the reduction of effective optical losses of the WM by 2.5%, thus reducing the required inversion of the QDs to maintain lasing and reducing the available gain for the SMs. This is a well-known effect from two-mode lasers in other setups^50–52^. In Fig. 6c, d, the corresponding simulated cross-correlation and optical spectra are depicted, matching the experimental data very well. In order to reproduce the conditions from Fig. 6b, simulations of the attenuated strong-mode spectra were superimposed onto the simulated WM spectra in Fig. 6d. Within the locking range of the WMs, intensity fluctuations are generally suppressed, thus leading to smaller delay peaks in the cross-correlation. At either edge of the locking range (*ν*_w_ ≈ ±1.5 GHz), the signature of the dynamic unlocking of both lasers becomes evident, leading to stronger peaks in the $g_{{\mathrm{w}}_1{\mathrm{w}}_2}^{(2)}$ cross-correlation. Depending on the detuning, the cross-correlation peak ±*τ* can be enhanced, i.e., the role of the leader in the leader-laggard synchronization of the microlasers is mainly taken on by the laser that is positively frequency-detuned with respect to the other laser. This asymmetry in the frequency detuning is due to the amplitude-phase coupling, i.e., nonzero *α*^12^. In Supplementary Note 4, we discuss the impact of the *α*-factor in more detail and compare experimental locking results with simulations considering a constant *α*-factor. An enhancement of the weak-mode correlations can be observed also within the locking range of the SMs, as the WMs are suppressed and driven further toward thermal (bunched) emission. For scenarios where strong correlation between the coupled laser emission is required, a detuning near the locking boundaries of the WMs or within the SM locking range should be preferred.

### Zero-lag synchronization of microlasers with self-feedback

Previous work showed the possibility of zero-lag synchronization of chaotic intensity fluctuations in small networks of mutually coupled semiconductor lasers, in particular if the lasers are also subject to feedback^14^. We explore this important regime of coupled nonlinear oscillators. Noteworthy, this setting (see Fig. 1b) in the single-photon regime could eventually be linked to entanglement of mutually coupled quantum systems^14^.

**Fig. 7:**
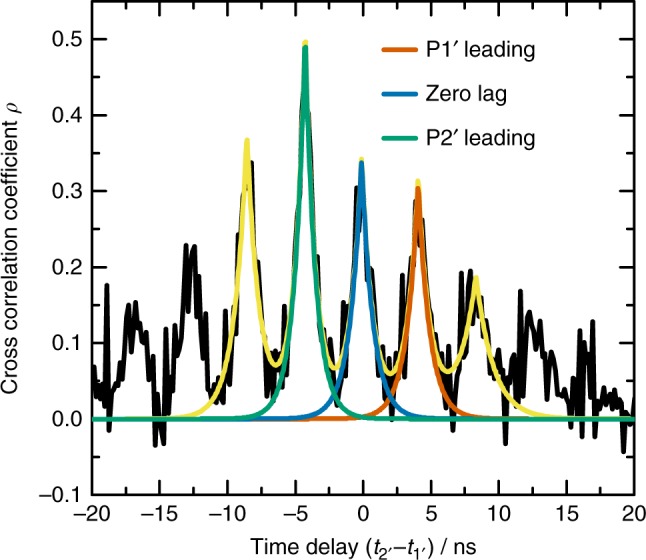
Cross-correlation coefficient of synchronized micropillar lasers. The plot shows the delay-dependent cross-correlation coefficient *ρ*(*τ* = *t*_2′_−*t*_1′_) of mutually coupled micropillar lasers with an additional mirror relay. In the delay range [−10 ns, 10 ns] the sum (bright yellow curve) of five peaks of the form *A* exp (−|*τ*−*τ*_center_|/*τ*_corr_) are fitted to the data (black). The zero-lag peak is depicted in blue, the leader-laggard peaks where pillar 1′ or pillar 2′ is leading are depicted in red and green, respectively

Here, we explore the possibility of zero-lag synchronization by introducing a mirror relay in the center of the beam path between the two oscillators. The length of the feedback beam path is chosen to introduce additional passive optical feedback to each cavity-enhanced microlaser. The feedback delay is equal to the coupling delay time between the pillars. A semipermeable mirror is thus placed at half distance in the coupling path, such that it introduces feedback of the required time delay. As seen in the previous discussion of Fig. 6, a strong cross-correlation between the coupled lasers can be expected in regions of dynamical instabilities. We therefore choose two other micropillar lasers, P1′ and P2′ (see Supplementary Notes 6 and 7 for more details), from the same arrays and couple them with a semipermeable mirror in the aforementioned setup. These pillars show a crossing of their SM and WM intensity in their current dependence at pump currents far above threshold and exhibit more frequent mode-switching events between their respective SM and WMs^43^. The SM competition at this operating point results in a striking increase in the autocorrelation $g_{{\mathrm{w}}_i{\mathrm{w}}_i}^{(2)}(0)$ and an enhanced sensitivity with respect to optical feedback^31^, which should enhance the correlation signatures when coupling the two microlasers. In order to quantify the cross-correlation $g_{{\mathrm{w}}_{1\prime}{\mathrm{w}}_{2\prime}}^{(2)}(\tau)$, we calculate the linear intensity cross-correlation coefficient for the two coupled pillars

3 $$\rho (\tau ) = \frac{{g_{{\mathrm{w}}_{1\prime}{\mathrm{w}}_{2\prime}}^{(2)}(\tau) - 1}}{{\sqrt {\left( {g_{{\mathrm{w}}_{1\prime}{\mathrm{w}}_{2\prime}}^{(2)}(0) - 1} \right)\left( {g_{{\mathrm{w}}_{1\prime}{\mathrm{w}}_{2\prime}}^{(2)}(0) - 1} \right)}}},$$

and expect a value of 1 (−1) for fully linearly correlated (anti correlated) dynamics and a value of 0 for uncorrelated dynamics. The resulting time-dependent correlation coefficient is displayed in Fig. 7. With the passive optical feedback from the micropillars to the semipermeable mirror, additional correlation peaks at a time delay of zero appear (blue line) if compared to Fig. 5a, along with revival peaks after integer multiples of the coupling delay. While the cross-correlation measurement shows zero-lag correlation coefficients of up to 34%, a strong peak of up to 50% at the coupling delay time (red and green lines) can be seen, corresponding to simultaneously occurring leader-laggard type synchronization. The coexistence of both zero-lag and leader-laggard synchronization peaks in the cross-correlation of the high-*β* microlasers can be interpreted as a coexistence or stochastic transition between the two types of dynamics. In that direction, strong noise is known to perturb coupled lasers away from the synchronization manifold^53^, leading to intermittent desynchronization events known as bubbling.

## Discussion

Synchronization of coupled systems is at the heart of nonlinear dynamics and can lead to a plethora of dynamical patterns ranging from leader-laggard behavior to zero-lag synchronization. It plays a vital role in our brain activity and can be applied for secure data communication. We set out to push the field of optical synchronization toward the few-photon regime by studying the joint dynamics of mutually coupled microlasers with cavity-enhanced functionality and sub-µW output powers. In our microlasers, spontaneous emission couples orders of magnitude more efficiently to the lasing mode compared to semiconductor lasers. Thus, their dynamics is crucially influenced by enhanced spontaneous emission noise which is negligible in the classical counterparts but plays an important role in our studies. Indeed, we merged the topical areas of nanophotonics and nonlinear physics by mutually coupling quantum-dot microlasers with a small intracavity photon number (<20), similar to numbers observed in micropillar lasers very recently by means of photon-number resolving detectors^54^, to explore the noise-governed regime of synchronization for the first time.

When our microlasers are not too far detuned, clear mutual locking of their emission frequencies is observed. Due to the high-spontaneous emission noise in the cavity-enhanced micropillar lasers, the locking remains imperfect, manifesting itself in a deviation of the locking slopes of both lasers. This behavior is in striking contrast to macroscopic coupled laser setups, where the unlocking transition is abrupt. The time-resolved intensity cross-correlation measurements show a partial synchronization of the intensity patterns, reaching correlation coefficients of up to 50%. When coupled with a passive relay, signatures of both zero-lag synchronization as well as leader-laggard type can be observed.

Our experimental results are described in excellent agreement by numerical simulations based on semiclassical rate equations. The simulations support the interpretation of noise-driven dynamics in our coupled system of cavity-enhanced optical oscillators and reveals a fine structure of the optical spectra of the locked microlasers comprising several compound laser modes, forming a frequency comb with a broad Gaussian envelope. We interpret this mode structure as a stochastic switching between different compound laser modes, which are individually locked between the coupled microlasers. A detailed experimental verification of this predicted behavior is subject to future work.

Noteworthy, the investigated QD microlasers exhibit charge carrier lifetimes in the order of 0.1 ns due to the enhancement of the spontaneous recombination rate and the carrier-density dependent scattering processes. Consequently, the carrier lifetime differs from the photon lifetime of 0.01 ns only by a factor of ten, which, compared to macroscopic quantum well lasers, is very low. The microlasers thus exhibit behaviour similar to class-A lasers, encompassing strongly damped relaxation oscillations and also a higher stability to external feedback^55,56^. Most of the existing literature on coupled lasers is devoted to the quantum-well laser case and thus new coupled dynamics might occur in our case. A theoretical bifurcation analysis for delay coupled lasers in^57^ already suggest that a smaller time-scale ratio leads to much wider locking ranges and new phase locked solutions, however, experimental results in this regime are still missing. Thus, besides the influence of stochastic effects on the dynamics, the strongly damped internal dynamics also plays a crucial role for the observed locking dynamics.

In summary, our experiments prove mutual coupling and zero-lag synchronization in the few-photons regime of interacting optical oscillators. We have revealed that in this regime with on the order of ten intracavity photons and high *β*-factors quantum noise starts to become significant and classical synchronization features get smeared out. We confirm these effects by highly sensitive single-photon cross-correlations. Interestingly, the issue of realizing synchronization in noise quantum systems has recently been explored theoretically and it was shown that it can be overcome by application of squeezing-driven oscillators^58^. As such the present experimental and theoretical results have high potential to open up new perspectives to explore synchronization at the crossroad between classical and quantum physics. Especially interesting in this sense would be investigations on mutual coupling of nanoscale oscillators, where the fascinating boundaries between classical synchronization and quantum entanglement phenomena^24,25,59^ can be experimentally explored.

## Methods

### Sample technology and experimental setup

The microlasers under study are 5 μm diameter electrically contacted micropillars based on AlGaAs heterostructures consisting of a single layer of In_0.3_Ga_0.7_As QDs with a density of 5 × 10^9^ cm^−2^ enclosed by two high-quality AlAs/GaAs distributed Bragg reflectors (DBR) (see Supplementary Note 1 for more technological details). This configuration ensures a small mode volume and pronounced light–matter interaction that result in cQED-enhanced coupling of spontaneous emission into the lasing mode^60^.

Using advanced nanofabrication technology and an optimized sample design we realized dense arrays of 120 QD-micropillars each. For the coupling experiments sample pieces each containing one of these arrays were placed into two independent He-flow cryostats separated by 700 mm and operated in a temperature range between 31 and 36 K. The lasers in the two selected arrays stem from neighboring parts of the same semiconductor wafer to ensure similar emission characteristics. All micropillars in the array share one common gold contact bar and are thus driven in parallel. Therefore, we chose voltage-driven operation (instead of the commonly preferable current-driven operation), in order to decouple the operating point of each micropillar from random electrical changes in other micropillars. The electrical current through each micropillar under investigation was estimated to be 1/120 of the current through the corresponding 120 micropillar array, but the exact current through a specific micropillar is not known

**Fig. 8:**
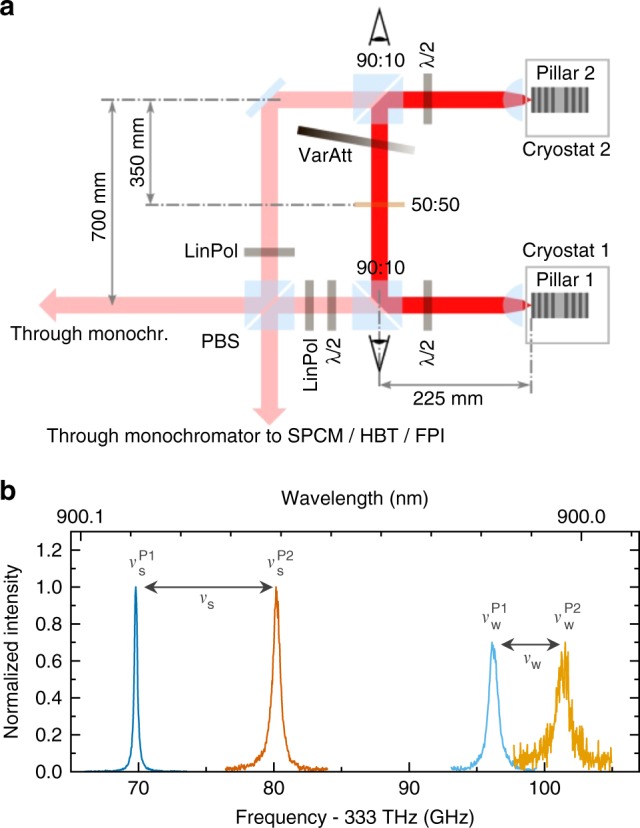
Experimental setup and high-resolution emission spectra. **a** Experimental setup showing the coupling beam path (solid red) and the detection beam paths (pale red). Each micropillar laser sample is placed in a cryostat at temperatures of *T*_1_ = 32 K and *T*_2_ ∈ [32 K, 36 K]. Linear polarizers (LinPol) in combination with half-wave plates (*λ*/2) are used for mode selection, a variable attenuation (VarAtt) is used to control the mutual coupling strength, and a 50/50 polarizing beam splitter (PBS) directs the pillar emission to the monochromators with attached detectors, which include single-photon counting modules (SPCM) that are used to measure high-resolution spectra by a Fabry–Perot interferometer (FPI) and intensity auto-correlations in a Hanbury Brown and Twiss (HBT) configuration or cross-correlations. In addition, 90/10 (90:10) beam splitters are used for white light illumination and monitoring of the sample surface. **b** Fabry–Perot interferometer (FPI) spectra of the noncoupled micropillar lasers P1 and P2. The strong mode and weak mode (respectively normalized to 1 and to 0.7) are depicted in the same color-coding as used in Fig. 2

Figure 8a presents the experimental setup which is used to study the mutual coupling of micropillar lasers via symmetric paths. Emission of each microlaser is first collimated by an aspheric lens with significantly reduced transmission losses if compared to usually used long working-distance microscope objectives and is then directed by beam-splitters with 90% reflectivity to the other microlaser of the selected pair. We would like to note that the use of an aspheric lens is crucial to achieve a high enough optical power level for the mutual coupling experiments between the microlasers. The transmitted light (10%) is directed via a PBS toward the two detection paths. Using polarization optics, it is possible to independently select the micropillar modes (strong and weak) being coupled and also those being detected. An optional variable attenuator (VarAtt) in the coupling path enables control of the coupling strength. In an alternative version of the experiment, which aims at the demonstration of zero-lag synchronization, the variable attenuator is substituted by a passive relay (pellicle mirror with 50% transmittance) placed in the center of the beam path between both pillars. The required submicrometer mechanical stability of the coupling beam path between the pillars with microscale upper facets is ensured by a customized video control loop in which the microscopic image of each sample was constantly monitored by a computer such that (unavoidable) temperature-induced sample shifts were automatically compensated by tracking the motorized linear stages of the corresponding cryostat.

A sample spectrum of the emission modes of the selected pair of micropillar lasers is shown in Fig. 8b. The diagram illustrates the definition of the nominal detuning $\nu _{\mathrm{s}} = \nu _{\mathrm{s}}^{{\mathrm{P2}}} - \nu _{\mathrm{s}}^{{\mathrm{P1}}}$ and $\nu _{\mathrm{w}} = \nu _{\mathrm{w}}^{{\mathrm{P2}}} - \nu _{\mathrm{w}}^{{\mathrm{P1}}}$ of the SM and WMs, respectively. Due to a different frequency splitting between strong and WMs in the two lasers, the SM and WM can be precisely and independently tuned in and out of resonance individually by centikelvin temperature changes. We would like to note that the SM–WM splitting of each individual pillar depends mainly on structural asymmetries and was found to be independent of temperature and injection current. This can also be seen in Fig. 2 (panels **c** and **d**), showing the pump current dependence of the individual mode frequencies. In laser P1 (P2), a splitting of 26 GHz (21 GHz) is found. Consequently, the nominal detunings of WMs and SMs of the selected micropillar lasers differ by a constant value of 5 GHz, *ν*_s_ = *ν*_w_ + 5 GHz. By changing the injection current and thus, the output intensities of the two microlasers the coupling configuration can be continuously tuned from a master/slave scenario (where the output power of one laser is much larger and drives the weak laser) to a mutual coupling scenario (where the output powers of both lasers are similar)^57^.

### Theoretical model

The model used in this paper is based on semiclassical stochastic rate equations^61^ taking into account the electron scattering mechanisms into the QDs as derived in our previous works^43,62,63^. The description of micro- and nanolasers with semiclassical equations was recently shown to be valid down to a surprisingly low number of emitters on the order of ten^64^. Our chosen theoretical framework should therefore be suited to accurately describe the dynamical properties of the micropillar lasers considered here. In our model we account for the two orthogonal linearly polarized micropillar modes by two separate complex electric field equations, denoted as WM and SM, corresponding to their respective output power above threshold as discussed in the previous section. As the microlaser output is predominantly linearly polarized and dominated by strong spontaneous emission, we couple both laser modes to a single-charge carrier type and describe the mode interaction by phenomenological gain compression terms. We thus neglect spin-flip dynamics required to model the behaviour in lower-*β* VCSEL devices^65,66^. For each of the two coupled lasers, we model the electrical fields *E*_j_ of the two modes *j* ∈ w, s, the occupation probability of the active and inactive QDs *ρ*_(in)act_, and the reservoir carrier density *n*_r_. Here we denote as active the portion of QDs within the inhomogeneous distribution that couple to the lasing mode via stimulated emission.

4 $$\begin{array}{*{20}{l}} {\frac{{\mathrm{d}}}{{{\mathrm{d}}t}}E_{\mathrm{j}}(t)} \hfill & = \hfill & {\left[ {{\textstyle{{\hbar \omega Z^{{\mathrm{QD}}}} \over {\epsilon _0\epsilon _{{\mathrm{bg}}}V}}}g_{\mathrm{j}}(2\rho _{{\mathrm{act}}}(t) - 1) - \kappa _{\mathrm{j}}} \right](1 + i\alpha )E_{\mathrm{j}}(t)} \hfill \\ {} \hfill & {} \hfill & { + \frac{\partial }{{\partial t}}E_{{\mathrm{j}}}{j}|_{{\mathrm{sp}}} + \frac{\partial }{{\partial t}}E_{\mathrm{j}}|_{{\mathrm{coup}}}} \hfill \end{array}{,}$$

5 $$\begin{array}{*{20}{l}} {\frac{{\mathrm{d}}}{{{\mathrm{d}}t}}\rho _{{\mathrm{act}}}(t)} \hfill & = \hfill & { - \mathop {\sum}\limits_{j \in \{ {\mathrm{s}},{\mathrm{w}}\} } {g_{\mathrm{j}}} [2\rho _{{\mathrm{act}}}(t) - 1]\left| {E_{\mathrm{j}}(t)} \right|^2 - \frac{{\rho _{{\mathrm{act}}}(t)}}{{\tau _{{\mathrm{sp}}}}}} \hfill \\ {} \hfill & {} \hfill & {S^{{\mathrm{in}}}n_{\mathrm{r}}(t)[1 - \rho _{{\mathrm{act}}}(t)]} \hfill \end{array}{,}$$

6 $$\begin{array}{*{20}{l}} {\frac{{\mathrm{d}}}{{{\mathrm{d}}t}}n_{\mathrm{r}}(t)} \hfill & = \hfill & {{\textstyle{\eta \over {e_0A}}}(J - J_{\mathrm{p}}) - S^{{\mathrm{in}}}n_{\mathrm{r}}(t){\textstyle{{2Z^{{\mathrm{QD}}}} \over A}}[1 - \rho (t)]} \hfill \\ {} \hfill & {} \hfill & { - \frac{{n_{\mathrm{r}}(t)}}{{\tau _{\mathrm{r}}}} - \frac{{2Z_{{\mathrm{inact}}}^{{\mathrm{QD}}}\rho _{{\mathrm{inact}}}}}{{A\tau _{{\mathrm{sp}}}}}} \hfill \end{array}{.}$$

The laser is pumped by injecting an electric current *J* into the reservoir *n*_r_ from where electrons may either recombine without contributing to the lasing mode or scatter into QDs with the rate *S*^in^ × *n*_r_(*t*). We account for experimental details in the pumping process by assuming a laser dependent injection efficiency *η* (see *η*^P1(P2)^ in Table 1), and a parasitic current *J*_p_, determined from fits to the experimental input–output curves, see also Fig. 2 and Table 1. The occupation of inactive dots is calculated from the steady-state value of Eq. (5) without stimulated emission, taking into account only spontaneous recombination within these dots:

7 $$\rho _{{\mathrm{inact}}}(t) = (\tau _{{\mathrm{sp}}}S^{{\mathrm{in}}}n_{\mathrm{r}})(1 + \tau _{{\mathrm{sp}}}S^{{\mathrm{in}}}n_{\mathrm{r}})^{ - 1}{.}$$

The electric fields of WM and SM both interact with the active QDs by stimulated emission. Since the frequencies of the two modes differ by only a few tens of μeV, we consider only one carrier population that is interacting with both optical modes, which leads to gain competition, modeled as

8 $$g_{\mathrm{j}} = g_{\mathrm{j}}^0\left( {1 + \varepsilon _0n_{{\mathrm{bg}}}c_0\mathop {\sum}\limits_{i \in \{ {\mathrm{s,w}}\} } {\varepsilon _{{\mathrm{ji}}}} |E_{\mathrm{i}}(t)|^2} \right)^{ - 1}{.}$$

The gain *g*_s,w_ of strong and WMs respectively depends on the individual intensity of both modes and the compression factors *ε*_ij_ with *i*, *j* ∈ {w, s}. A mode with high intensity reduces (compresses) the gain for both modes.

Spontaneous emission into the lasing modes is modeled via a Gaussian white noise source $\xi (t) \in {\Bbb C}$, where 〈*ξ*(*t*)〉 = 0 and 〈*ξ*(*t*)*ξ*(*t*′)〉 = *δ*(*t* − *t*′), such that

9 $$\frac{\partial }{{\partial t}}E_{\mathrm{j}}|_{{\mathrm{sp}}} = \sqrt {\beta \frac{{\hbar \omega }}{{\varepsilon _0\varepsilon _{{\mathrm{bg}}}}}\frac{{2Z^{{\mathrm{QD}}}}}{V}\frac{{\rho _{{\mathrm{act}}}^2}}{{\tau _{{\mathrm{sp}}}}}} \xi (t).$$

We simulate the two coupled micropillar lasers each with its own set of differential Eqs. (4)–(6), with the two lasers indicated by an index P1, P2, respectively. In the rotating frame of the free-running emission frequency of P2, the mutual coupling of the two lasers is expressed by

$$\begin{array}{*{20}{l}} {\frac{\partial }{{\partial t}}E_{\mathrm{j}}^{{\mathrm{P1}}}|_{{\mathrm{coup}}}} \hfill & = \hfill & {K\kappa _{\mathrm{j}}^{{\mathrm{P1}}}E_{\mathrm{j}}^{{\mathrm{P2}}}(t - \tau ) + 2\pi i\nu _{\mathrm{j}}E_{\mathrm{j}}^{{\mathrm{P1}}},} \hfill \\ {\frac{\partial }{{\partial t}}E_{\mathrm{j}}^{{\mathrm{P2}}}|_{{\mathrm{coup}}}} \hfill & = \hfill & {K\kappa _{\mathrm{j}}^{{\mathrm{P2}}}E_{\mathrm{j}}^{{\mathrm{P1}}}(t - \tau ),} \hfill \end{array}$$

where *K* is the coupling strength and *τ* the time delay after which the light from one laser arrives at the other. The term *ν*_s_ accounts for the relative frequency detuning between the two SMs, with an additional 5 GH detuning between the WMs due to the mode splitting mentioned above: *ν*_w_ = *ν*_s_ + 5 GHz.

Using the above model, we can accurately reproduce the measured input–output characteristics and current-dependent linewidths (see lines in Fig. 8), and allows for an accurate extraction of model parameters from the measured data. The slight differences in the laser characteristics between the two microlasers lead also to slightly different input parameters for the modeled devices. The parameters used in the simulations are listed in Table 1.

### Description of locking slopes by coupled phase oscillators description

To theoretically analyze our experimental and numerical observations in Fig. 4b), we reduce our laser model to a system of coupled phase oscillators^4^. We do so by neglecting the amplitude dynamics of the electric fields within the microlasers and setting the linewidth enhancement factor *α* = 0. Dropping the Henry factor *α* is required to obtain clear analytical solutions. The resulting phase equations read

$$\begin{array}{*{20}{l}} {\dot \varphi _1(t)} \hfill & = \hfill & {\varepsilon _{2 \to 1}{\mathrm{sin}}(\varphi _2(t - \tau ) - \varphi _1(t))} \hfill \\ {\dot \varphi _2(t)} \hfill & = \hfill & {\varepsilon _{1 \to 2}{\mathrm{sin}}(\varphi _1(t - \tau ) - \varphi _2(t)) + 2\pi \nu } \hfill \end{array}{.}$$

In order to quantify the locking dynamics, we define the locking slope *m*

$$m = \frac{{{\mathrm{d}}f}}{{{\mathrm{d}}\nu }}{\kern 1pt} ,$$

where $2\pi f = \dot \varphi _1 = \dot \varphi _2$ is the common phase velocity of the mutually locked oscillators. A locking slope of *m* = 0 or *m* = 1 denotes the limit cases where the locked oscillation frequency of both oscillators is given by the free-running frequency of oscillator 1 or 2, respectively.

Within this approach, the locking slope *m* depends on the quotient of the coupling strengths

$$\varepsilon _{{\mathrm{n \to m}}} = K\kappa _{\mathrm{j}}^{{\mathrm{P}}{\mathrm{m}}}\left| {\frac{{E_{\mathrm{j}}^{{\mathrm{P}}{\mathrm{n}}}}}{{E_{\mathrm{j}}^{{\mathrm{P}}{\mathrm{m}}}}}} \right|{\kern 1pt} ,$$

and can be calculated approximately to

10 $$m_{{\mathrm{P2}}}^{ - 1} - 1 \approx \frac{{\varepsilon _{1 \to 2}}}{{\varepsilon _{2 \to 1}}} + 2\tau {\kern 1pt} \varepsilon _{1 \to 2}.$$

The second term on the right hand side dominates the locking slope for all cases considered in this work.

## Supplementary information

Supplementary Information

## Data Availability

The data supporting the findings presented in this study are available from the corresponding author upon request.
