# Supplementary material for: Mutual coupling and synchronization of optically coupled quantum-dot micropillar lasers at ultra-low light levels
Source: Nat Commun. 2019 Apr 4;10:1539. doi: 10.1038/s41467-019-09559-2 (PMC6449346; doi:10.1038/s41467-019-09559-2)
Supplement: Supplementary file 1 — Supplementary Information [file 41467_2019_9559_MOESM1_ESM.pdf]

Supplemental material:
Mutual coupling and synchronization of optically coupled quantum-dot micropillar lasers at ultra-low light levels

Kreinberg et al.

SUPPLEMENTARY NOTE 1: SAMPLE AND EXPERIMENTAL METHODS

The micropillar lasers under study are based on AlGaAs heterostructures grown by molecular beam epitaxy. They contain a single active layer of $In_{0.3}Ga_{0.7}As$ quantum dots with a density of $5 \times 10^9\,\mathrm{cm}^{-2}$ inside a planar one-λ microcavity. This cavity is enclosed by two high-quality AlAs/GaAs distributed Bragg reflectors (DBR) to ensure a small mode volume and enhanced light-matter interaction. Two dense arrays of 5 µm diameter micropillar lasers with a pitch of 30 µm are realized by high-resolution electron-beam lithography followed by reactive-ion etching. Planarization with benzocyclobutene (BCB) protects the AlAs layers from oxidizing and provides mechanical support to the ring-shaped upper gold contacts. Each fabricated array includes 120 electrically driven micropillar lasers emitting in the spectral range 898 - 906 nm. For a more detailed description on the fabrication of electrically contacted micropillars we refer to Ref. [1]. Each array is placed on the cold finger in the vacuum chambers of its own dedicated helium flow cryostat, where the microlasers are operated at cryogenic temperatures (31 K - 36 K). For high spatial resolution, the market provides so called "microscopy cryostats" in fry pan geometry. These devices offer high mechanical stability, a short distance between sample and window, and a big window aperture for high-NA microscopy and micro-photoluminescence (µPL) spectroscopy. Moreover, they allow for electrical contacting the laser arrays and for an easy and safe exchange of the rather delicate wire bonded samples.

The mutual coupling experiments are very challenging and require finding two micropillar lasers with close to identical optical properties in terms of emission wavelength and input-output characteristics, which must remain constant over long integration times and many days of measurements. Therefore, pairs of lasers were selected which differ by no more than 1 nm in emission wavelength. In this context it is interesting to note that the rather delicate ring-shaped contact design with lateral current injection is very sensitive to temperature changes in cool-down cycles which can irreversibly change the input-output characteristics. Similarly, long-term experiments at high injection currents can also lead to degradation effects. Moreover, the output power of both microlasers needs to be high enough to enable sufficiently high mutual coupling strengths. As it turned out, achievable output powers of a few hundred nW are just high enough to demonstrate coupling effects even in an experimental setup, that is elaborately optimized to minimize losses in the coupling path. The chosen micropillar lasers emit at a wavelength of 900 nm with output powers up to 300 nW.

Two distinct pump scenarios have been chosen for pillar 1 (P1): $V_{\mathrm{bias,P1}} = 8.1\,\mathrm{V}$ ($I_{\mathrm{P1}} = 23.4\,\mu\mathrm{A}$ and $P_{\mathrm{out,P1}} = 99\,\mathrm{nW}$) and $V_{\mathrm{bias,P1}} = 8.5\,\mathrm{V}$ ($I_{\mathrm{P1}} = 27.2\,\mu\mathrm{A}$ and $P_{\mathrm{out,P1}} = 237\,\mathrm{nW}$). Meanwhile its temperature was always kept constant at 32 K. For each P1 pump scenario, several pillar 2 (P2) supply voltage steps where investigated. For every resulting voltage combination, the emission frequency of P2 was scanned over the emission frequency of P1 by sweeping the temperature of P2. During this continuous tuning process, spectra of each micropillar were acquired using a Fabry-Perot scanning interferometer (FPI) with a FWHM resolution of 120 MHz. In this context it is important to distinguish on the one hand the nominal frequencies ν_1, ν_2 of P1 and P2, respectively, and on the other hand the actually detected frequency f_{FPI}. The nominal frequencies ν_1, ν_2 are defined as the maxima of the rather Lorentzian shaped spectral power densities $S_1(f_{\mathrm{FPI}})$, $S_2(f_{\mathrm{FPI}})$ of each pillar, under the (hypothetical) condition, that each pillar is operated isolated. The temperature-controlled nominal detuning of the two micropillars is therefore given by $\nu = \nu_2 - \nu_1$. Further it is convenient not to use the absolute frequency f_{FPI} to describe the measured spectrum, but to use the frequency difference to the nominal frequency of pillar 1 (ν_1, yielding the relative frequency $f = f_{\mathrm{FPI}} - \nu_1$. Supplementary Figure 1 shows an extended schematic representation of the experimental setup which is used to study the mutual coupling of micropillar lasers. In comparison to the schematic in the main manuscript, Fig. 8(a), it gives additional information on the detection of the laser light. For monochromatic filtering and for acquiring overview spectra (using Peltier-cooled CCD detectors), the microlaser emission is sent through Czerny-Turner grating spectrometers with 750 mm focal length. The light transmitted through each monochromator is coupled into a multi-mode fiber for Si-avalanche photodiode-based time correlated single photon counting. Part of the filtered light is not coupled into a multi-mode fiber but into a single-mode fiber for spectral analysis by a confocal scanning Fabry-Perot interferometer (FPI) with a free spectral range of 7.5 GHz. The duration of one FPI scan is approximately 1-2 seconds.

SUPPLEMENTARY NOTE 2: COHERENCE TIMES OF MUTUALLY COUPLED MICROLASERS

The optical coupling of two semiconductor lasers was previously shown to improve the coherence properties of both lasers by suppressing the noise-induced phase drift [2–5]. The high β-factor of the microlasers makes them a strongly noise-dominated system. Investigating the dependence of the noise and coherence properties of the coupled microlasers on the control parameters is thus an important aspect of our studies on the cavity-enhanced mutually coupled oscillators. To explore the underlying physics, we extract the coherence times of the coupled microlasers from their spectral linewidth, both in the locked region for small detuning and for unlocked lasers with a detuning of $\nu_s = \pm 3\,\mathrm{GHz}$, as shown in Supplementary Figure 2. We evaluate the coherence time dependence on the output power

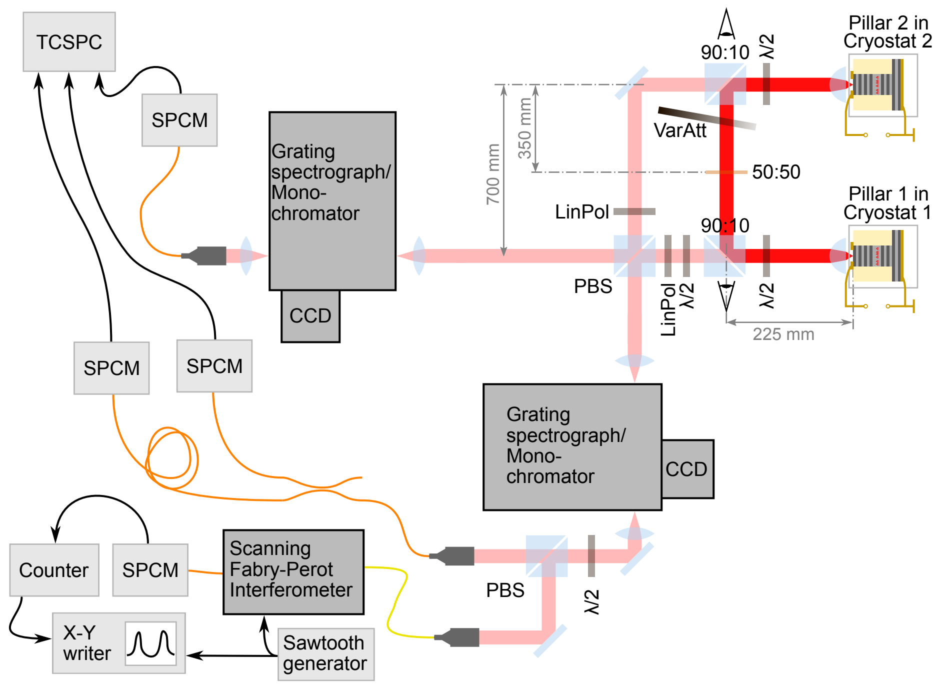

Supplementary Figure 1. Extended schematic of the experimental setup. The setup includes the coupling beam path (solid red) and the detection beam paths (pale red). Each micropillar laser sample is placed in a cryostat at temperatures of $T_1 = 32\,\mathrm{K}$ and $T_2 \in [32\,\mathrm{K}, 36\,\mathrm{K}]$. The micropillar lasers are electrically driven under cw conditions. Single-photon counting modules (SPCM) are used to measure auto-correlations in a Hanbury Brown and Twiss (HBT) configuration or cross-correlations via time correlated single photon counting (TCSPC). For acquiring high resolution spectra, the transition through a piezo-driven scanning Fabry-Perot interferometer is measured by counting the detection events from a SPCM. The spectra are calculated from the countrate dependence on the piezo voltage. Additional abbreviations: TCSPC - time correlated single photon counting, CCD - charge coupled device camera, LinPol - linear polarizer, VarAtt - variable attenuator, PBS - 50/50 polarizing beam splitter, $\lambda/2$ - lambda-half plate, 90:10 - beam splitter for white light illumination and monitoring of the sample surface, 50:50 - glass plate to realize symmetric self-feedback of mutually coupled micropillar lasers.

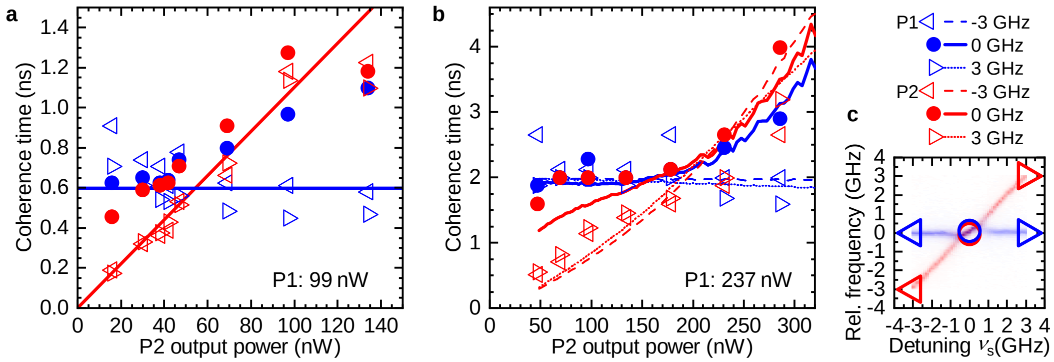

Supplementary Figure 2. Coherence times of the mutually coupled micropillar lasers. **a**, **b** Coherence times of coupled laser P1 and P2 vs. output power of laser P2 (blue: pillar P1, red: pillar P2). The coherence times are determined from the optical linewidths. **c** Example of the locking diagram to illustrate the detunings within ($\nu_s = 0$, circles) and outside the locking range ($\nu_s = \pm 3\,\mathrm{GHz}$ triangles) from where the coherence times are calculated. In addition, the blue and red line in panel **a** represent the mean free-running coherence time of P1 and fits of the Schawlow-Townes limit to the free-running coherence times of P2 (yielding a Q-factor of 50 000), respectively. The solid and dashed/dotted lines in panel **b** show the simulation results for the laser coherence time inside and outside of the locking range, respectively.

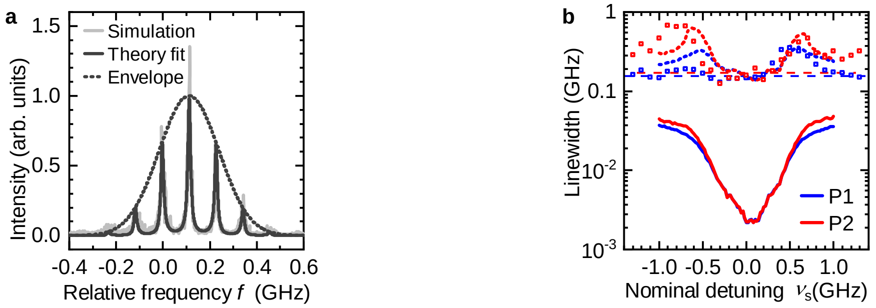

Supplementary Figure 3. Emission spectra and linewidths of the mutually coupled micropillar lasers. The lasers are driven with pump currents of $I_{P1} = 27.0\,\mu A$ and $I_{P2} = 24.0\,\mu A$. **a** Simulated spectrum of pillar P1 in a mutual coupling setup (light gray), along with a fit to the spectrum (dark gray) using a Fabry-Perot interferometer-like spectral transmission function with a Gaussian envelope (dashed gray). **b** Extracted linewidths of the Gaussian envelope (dotted line) and individual Fabry-Perot lines (solid line) for pillar P1 (blue) and P2 (red). As reference, the free-running linewidth is shown in dashed lines. Linewidths from fits to experimental spectra for $I_{P1} = 28.8\,\mu A$ and $I_{P2} = 24.6\,\mu A$ are plotted as open squares.

of pillar P2, while keeping the voltage of pillar P1 constant. This way, the coupling scheme can be tuned between master-slave-like coupling (low P2 power) and symmetric mutual coupling (equal power) of P1 and P2. The coherence times outside of the locking ranges can be seen to be only weakly influenced by the coupling, and just increase with increasing laser power. The coherence time of laser P1 stays constant outside of the locking range, as its output power stays constant. This indicates that a small detuning of 3 GHz between P1 and P2 only weakly influences the coherence properties of both lasers. Within the locking range (circles in Supplementary Figure 2), when the output power of pillar P2 is below that of pillar P1 (237 nW), a pronounced improvement of the coherence time of P2 towards that of P1 can be observed in the locking range. In contrast, for higher output power of P2, the coherence time of P1 is pulled towards that of P2. The coherence time of the mutually locked lasers is therefore determined predominantly by the stronger laser [6], which is also the laser with higher coherence time.

Interestingly, the numerical simulations reveal additional spectral features within the laser line. Supplementary Figure 3**a** illustrates the presence of a fine structure in the emission spectra. The spectra are composed of a regular frequency comb with $\approx 130\,\text{MHz}$ spacing, corresponding to the total round-trip coupling delay of $2\tau = 7.7\,\text{ns}$. We interpret the resulting spectral shape as a stochastic excitation of different compound laser modes (CLMs), i.e., standing waves within the combined cavity formed by the coupled micropillar lasers [7]. The stochastic switching between different CLMs leads to the presence of many different spectral peaks, weighted with a Gaussian envelope function [8]. Experimentally, this fine structure cannot be resolved due to insufficient spectral resolution of the FPI. In order to resolve the fine structure, a resolution of 50 MHz, preferably 10 MHz, would be required. For comparison with the experiment, the resulting numerical spectra must be convolved with an artificial Lorentzian-detector response function. Supplementary Figure 3**b** depicts the experimental and numerical (both raw and convolved) linewidth dependencies with respect to the nominal detuning between the two lasers. While the spectral width of the Gaussian envelope (dotted lines) - which is in good agreement with the experimental data (open squares) - is reduced only down to the free-running laser linewidth, the individual Fabry-Perot modes (solid lines) exhibit a strong narrowing inside the locking range. Their linewidths are reduced down to a few MHz. This indicates strong coherence within each of the compound laser modes.

At the locking boundaries, the width of the Gaussian envelope is observed to exceed the free-running laser linewidth. This is a signature of dynamical instabilities at the locking boundaries, leading to a strongly reduced coherence of the laser light output near the unlocking transition. The underlying bifurcation structure of the deterministic system is strongly washed out due to the noise-dominated nature of the high-β microlasers. In this highly stochastic regime, we therefore rely on spectral and correlation properties, which are presented in the main manuscript, in order to more comprehensively characterize the laser dynamics.

SUPPLEMENTARY NOTE 3: SLOPE OF THE LOCKING REGION FOR INSTANTANEOUSLY COUPLED PHASE OSCILLATORS

In addition to the analytical investigation of the locking slope of time-delayed phase oscillators in the main manuscript, we present here analytical calculations of the locking slope of instantaneously coupled phase oscillators.

We describe the two microlasers P1 and P2 as two phase-coupled oscillators with phases φ_1 and φ_2:

$$\dot{\varphi_1} = \omega_1 + \varepsilon_1 \sin(\varphi_2 - \varphi_1) \tag{1}$$
$$\dot{\varphi_2} = \omega_2 + \varepsilon_2 \sin(\varphi_1 - \varphi_2) \tag{2}$$

Considering the phase difference $\psi = \varphi_2 - \varphi_1$ and nominal detuning $\Delta\omega = \omega_2 - \omega_1$, the dynamics of the phase difference is described by

$$\dot{\psi} = \Delta\omega - (\varepsilon_1 + \varepsilon_2)\sin\psi. \tag{3}$$

Phase locking is achieved, if the phase difference is constant $\dot{\psi} = 0$ yielding

$$\sin\psi = \frac{\Delta\omega}{\varepsilon_1 + \varepsilon_2}. \tag{4}$$

The phase evolution of the two oscillators while locked:

$$\dot{\varphi_1} = \omega_1 + \frac{\varepsilon_1}{\varepsilon_1 + \varepsilon_2}\Delta\omega \tag{5}$$
$$\dot{\varphi_2} = \omega_2 - \frac{\varepsilon_2}{\varepsilon_1 + \varepsilon_2}\Delta\omega \tag{6}$$

We determine the locking slope m as the frequency change per nominal detuning change in the locking range. Since we keep ω_1 constant, this translates to the partial derivatives of the locked frequencies $\dot{\varphi_1}$, $\dot{\varphi_2}$ with respect to ω_2:

$$m = \frac{\mathrm{d}\dot{\varphi_1}}{\mathrm{d}\omega_2} = \frac{\varepsilon_1}{\varepsilon_1 + \varepsilon_2} = 1 - \frac{\varepsilon_2}{\varepsilon_1 + \varepsilon_2} = \frac{\mathrm{d}\dot{\varphi_2}}{\mathrm{d}\omega_2} \tag{7}$$
$$\Leftrightarrow m^{-1} = \frac{\varepsilon_1 + \varepsilon_2}{\varepsilon_1} = 1 + \frac{\varepsilon_2}{\varepsilon_1} \tag{8}$$
$$\Leftrightarrow m^{-1} - 1 = \frac{\varepsilon_2}{\varepsilon_1} \tag{9}$$

Since the coupling strength parameters ε_1 and ε_2 are proportional to the ratio of the injected field to the intracavity field

$$\varepsilon_1 \sim \frac{E_2}{E_1} \sim \sqrt{\frac{P_2}{P_1}} \tag{10}$$
$$\varepsilon_2 \sim \frac{E_1}{E_2} \sim \sqrt{\frac{P_1}{P_2}}, \tag{11}$$

it follows

$$m^{-1} - 1 \sim \frac{P_1}{P_2}$$

for the dependence of the locking slope on the output powers P_1 and P_2 of P1 and P2, respectively. As pointed out in the main manuscript, both experiment and simulation show a deviation of this prediction: The exponent of P_2 is not -1(as predicted here) or -0.5 (as predicted in the main manuscript), but rather -2.

For further analysis, the model from Eq. (2) in the main manuscript was fitted to numerical simulations with different coupling rates, see Supplementary Figure 4**a**. The exponents A and the difference in x-intercept through $m = 0.5$ are shown in Supplementary Figure 4**b**. The simulations predict that the difference in the locking slopes of the two lasers vanishes when the coupling strength K is increased. A transition from the observed partial locking towards a stronger phase locking is therefore possible by increasing the coupling rate.

SUPPLEMENTARY NOTE 4: LINEWIDTH ENHANCEMENT FACTOR

When considering the spectral position of the locked laser emission, we observe that at zero nominal detuning, the lasers are oscillating not at their free-running frequency, but are shifted to higher frequency (Supplementary Figure 5, panel **a** for pillar P1 and panel **b** for pillar P2). Furthermore, even the start and end nominal detunings of the locking

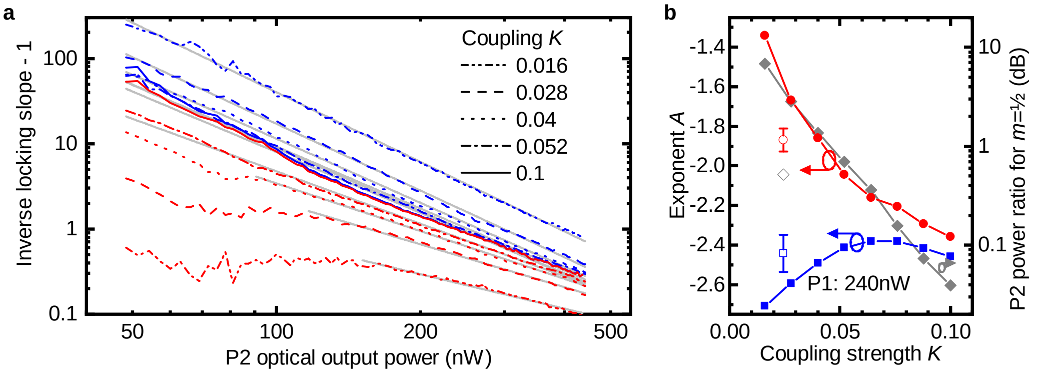

Supplementary Figure 4. Coupling strength-dependent study of locking slopes. **a** Simulated locking slopes in dependence of P2 output power for different coupling strengths K. The pump currents are $I_{\mathrm{P1}} = 27.1\,\mu\mathrm{A}$, $I_{\mathrm{P2}} = 24.4\,\mu\mathrm{A}$. The higher the coupling strength K, the more similar the locking slope. **b** Exponent A of locking slope-output power dependence (c.f. main text) of the two micropillar lasers as a function of the coupling strength K. The higher the coupling strength, the closer are the values of the exponents. The gray dots indicate the necessary increase in output power of P2 to go from $m_{\mathrm{P2}} = 0.5$ to $m_{\mathrm{P1}} = 0.5$ (indicated by the gray dotted line in Fig. 4**b** in the main manuscript).

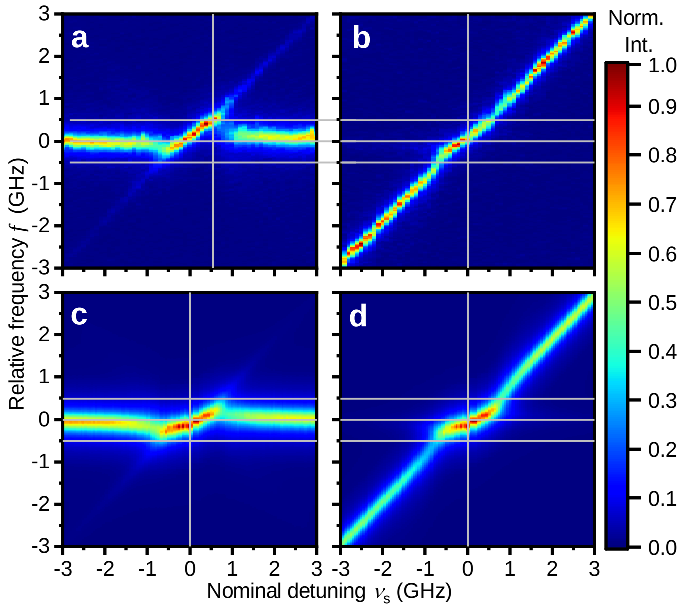

Supplementary Figure 5. Frequency shift by phase-amplitude coupling. **a, b** Emission intensity of pillar P1 (panel **a**) and pillar P2 (panel **b**) for injection currents of $I_{\mathrm{P1}} = 28.8\,\mu\mathrm{A}$, $I_{\mathrm{P2}} = 27.9\,\mu\mathrm{A}$, voltages of $V_{\mathrm{bias,P1}} = 8.5\,\mathrm{V}$, $V_{\mathrm{bias,P2}} = 8.7\,\mathrm{V}$ and output powers $P_{\mathrm{out,P1}} = 238\,\mathrm{nW}$, $P_{\mathrm{out,P2}} = 257\,\mathrm{nW}$. The experimental results demonstrate that the locking range is not symmetric to zero detuning. At zero nominal detuning the lasers are shifted to higher frequency. **c, d** Theoretical modelling for pillar P1 (panel **c**) and pillar P2 (panel **d**) with fixed phase-amplitude coupling factor α and for pump currents of $I_{\mathrm{P1}} = 27.1\,\mu\mathrm{A}$, $I_{\mathrm{P2}} = 24.4\,\mu\mathrm{A}$ and output powers $P_{\mathrm{out,P1}} = 238\,\mathrm{nW}$, $P_{\mathrm{out,P2}} = 134\,\mathrm{nW}$. The asymmetry of the locking range is reproduced in good agreement with experiment. But, in contrast to experiment, the emission frequency at zero detuning is negatively, not positively shifted. Moreover, the locking region is not represented by a straight line but by a curved line, which proves the assumption of a constant α factor to be inappropriate for QD micropillar lasers.

range are not symmetric to zero. We point out, that the former effect can only be observed in a mutual locking scheme, but not in an optical injection scheme. Further, it cannot be identified if nothing except a beating signal is available, instead the spectra of both lasers need to be analyzed independently. Both effects indicate phase-amplitude coupling, in semiconductor lasers usually described via the linewidth enhancement factor α[9].

In our numerical model we use a constant α-factor, obviously the locking range is shifted inversely as in the experiment. Moreover, and in contrast to the experimental results, the locking range is bent (Supplementary Figure 5, panel **c** for pillar P1 and panel **d** for pillar P2), indicating an overestimation of the phase-amplitude coupling effect

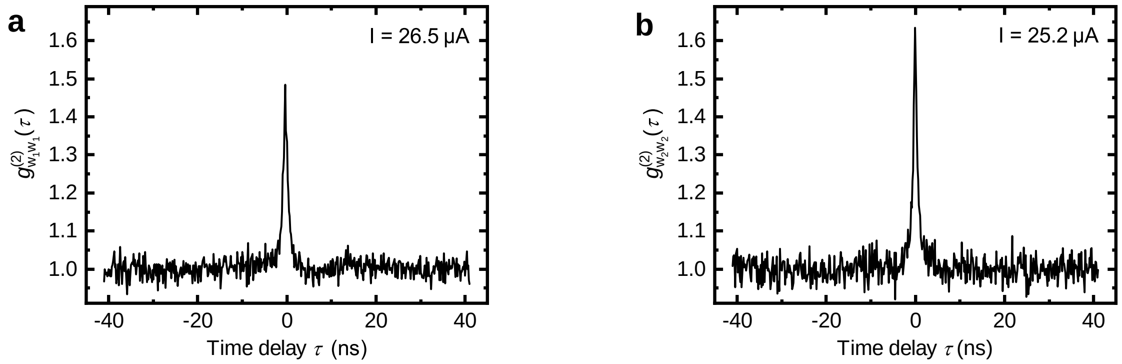

Supplementary Figure 6. Equal-time intensity auto-correlation functions of non-coupled micropillar lasers. The panels show the auto-correlation function $g^{(2)}_{\mathrm{w_1w_1}}(\tau)$ and $g^{(2)}_{\mathrm{w_2w_2}}(\tau)$ of the weak modes of P1 (panel **a**) and P2 (panel **b**), respectively. The photon bunching is attributed to mode switching events.[12]

in the center of the locking range. Interestingly, it has been shown that a constant α-factor fails to properly model quantum dot lasers and that the effective α-factor inside the locking range is smaller than on the edges of the locking range [10]. This could explain the overestimation of the phase-amplitude coupling effect in the center of the locking range. What is not explained up to now is the fact that the frequency of the coupled pillars at zero detuning is shifted to higher frequency. For a positive α-factor a negative shift is expected, as reproduced in the simulations. One possible explanation is that we drive the pillars constant-voltage, not constant current. Under optical injection, the voltage of current-driven VCSELs drops [11]. Since the micropillar lasers are voltage-driven, a voltage drop at the active region can only be compensated by higher DBR voltage, hence higher current. As long as current-induced heating can be neglected, an increased current would lead to the observed blue shift.

SUPPLEMENTARY NOTE 5: INTENSITY AUTO-CORRELATION OF MUTUALLY COUPLED MICROPILLAR LASERS

For putting into context the cross-correlation $g^{(2)}_{\mathrm{w_1w_2}}$ peak heights in Fig. 5 in the main manuscript, the intensity auto-correlations of the weak modes of P1 and P2 are shown for typical pump currents in Supplementary Figure 6. The pillars are not optically coupled to each other. The photon bunching indicated by $g^{(2)}(0) \approx 1.5$ is partly due to spontaneous emission, but mainly attributed to mode competition-induced mode switching events.[12]

SUPPLEMENTARY NOTE 6: DETUNING-INDEPENDENT INTENSITY OF MUTUALLY COUPLED MICROPILLAR LASERS

The question appears, whether mutual optical coupling increases the strong mode output power of the coupled QD micropillar lasers. As it turns out, the experimental data provides no evidence that the strong mode emission overall intensity of both pillars is significantly influenced by their locking, compare Supplementary Figure 7. However, locking of the strong modes strongly reduces the weak mode intensity and vice versa, as pointed out in Fig. 6 in the main manuscript.

SUPPLEMENTARY NOTE 7: OPTICAL PROPERTIES OF MUTUALLY COUPLED MICROPILLAR LASERS P1' AND P2'

As pointed out in the main manuscript, we chose a second pair of micropillar lasers from the same wafer, P1' and P2', which exhibit more chaotic dynamics. By doing so we aim for stronger cross correlation signals than the ones measured on pillars P1 and P2. These pillars show a crossing of their strong mode and weak mode intensity in their voltage dependence at voltages far above strong mode threshold, as shown in Supplementary Figure 8 a). The mode competition is very strong near this crossing voltage, resulting in a striking increase in $g^{(2)}(0)$ and an enhanced sensitivity with respect to optical feedback. [14] In order to put the cross-$g^{(2)}(\tau)$ of P1' and P2' into relation with

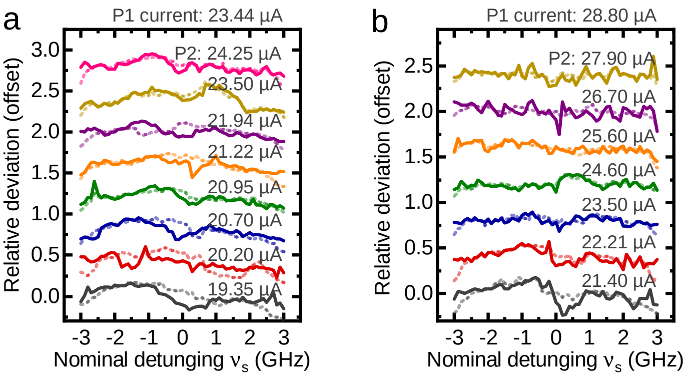

Supplementary Figure 7. Detuning-dependence of overall strong mode intensity. The data recorded were for an injection current of $I_{\mathrm{P1}} = 23.44\,\mu\mathrm{A}$ (panel **a**) and $I_{\mathrm{P1}} = 28.80\,\mu\mathrm{A}$ (panel **b**), respectively. Solid lines indicate fit results, dotted ines indicate results from full-spectrum integration. The data provides no evidence that locking of the strong modes influences their emission intensity.

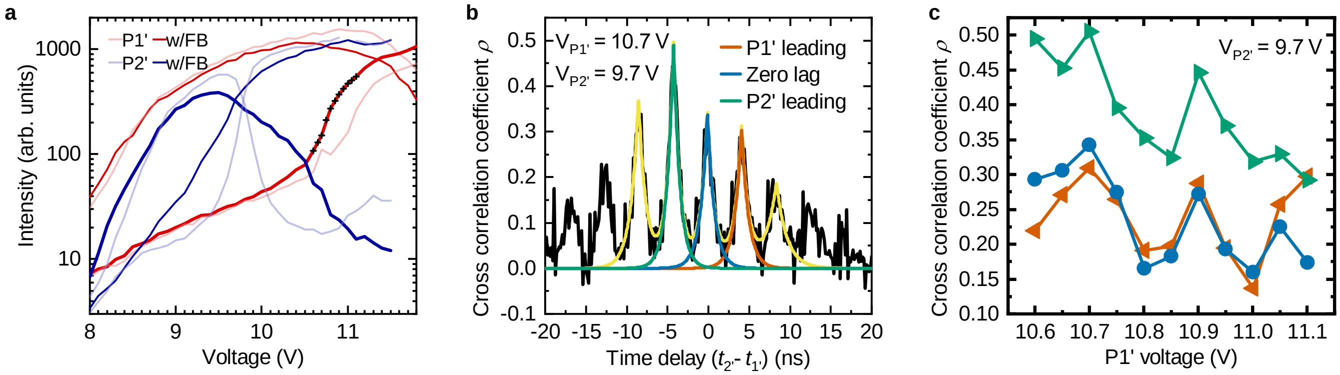

Supplementary Figure 8. Output and coupling properties of a second pair of micropillar lasers. **a** Voltage dependence of output intensity of the non-coupled pillars P1' and P2' (light colors). Dark colors depict measurements of the respective pillar with applied 50 % optical feedback, delayed by 3.85 ns. In following experiments, the thick plotted modes are coupled with a 50:50 mirror at half distance. **b** Delay-dependent intensity linear cross correlation coefficient $\rho(\tau)$ of coupled P1' and P2'. In the delay range $[-10\,\mathrm{ns}, 10\,\mathrm{ns}]$ the sum (bright yellow curve) of five peaks of the form $A\exp(-|\tau - \tau_{\mathrm{center}}|/\tau_{\mathrm{corr}})$ are fitted to the data (black). The zero-lag peak is depicted in blue, the leader-laggard peaks where P1' or P2' is leading are depicted in red and green, respectively. Since the blue peak is smaller than its green neighbour, dominant zero-lag synchronization has to be discarded. The correlogram is better understood by assuming two pillars exposing the feedback-typical revival peaks[13], which are then mutually relaying each other. **c** Amplitudes A of the zero-lag and leader-laggard peaks obtained from the cross-correlation coefficient diagrams as shown in b) for different voltages of P1'.

the auto-$g^{(2)}(0)$ of both pillars, we calculate the linear intensity cross correlation coefficient for P1' and P2'

$$\rho(\tau) = \frac{g^{(2)}_{\mathrm{w}_{1'},\mathrm{w}_{2'}}(\tau) - 1}{\sqrt{\left(g^{(2)}_{\mathrm{w}_{1'},\mathrm{w}_{1'}}(0) - 1\right)\left(g^{(2)}_{\mathrm{w}_{2'},\mathrm{w}_{2'}}(0) - 1\right)}}, \tag{12}$$

and expect a value of 1 (-1) for fully linearly correlated (anti correlated) dynamics and a value of 0 for uncorrelated dynamics. The results are displayed in Supplementary Figure 8(b), showing zero-lag correlation coefficients of up to 34 %. However, the dynamics is still of the leader-laggard type, and even unidirectionally dominated by P2' with correlation coefficients of up to 50 %. As described in the main manuscript, we obtained the cross-$g^{(2)}$ of P1' and P2' at delays which correspond to leader-laggard type or zero lag synchronization from the corresponding cross-correlogram by fitting a sum of peaks to the measured data. This is also illustrated in Supplementary Figure 8 b). We did so for several voltages of P1', which are indicated by crosses in Supplementary Figure 8 a), yielding a voltage dependence of the correlation peak height, shown in Supplementary Figure 8 c). For most voltages of P1', the synchronization is of the leader-laggard type and dominated by P2'. Symmetric leader-laggard synchronization is only observed for the highest applied P2' voltages, i.e. for 11.05 V and for 11.10 V.

SUPPLEMENTARY REFERENCES

[1] Böckler, C. *et al.* Electrically driven high-Q quantum dot-micropillar cavities. *Appl. Phys. Lett.* **92**, 091107 (2008).
[2] Agrawal, G. P. Line narrowing in a single-mode injection-laser due to external optical feedback. *IEEE J. Quantum Electron.* **20**, 468–471 (1984).
[3] Hegarty, S. P. *et al.* Phase-locked mutually coupled 1.3 um quantum-dot lasers. *Opt. Lett.* **32**, 3245–3247 (2007).
[4] Brunner, D., Luna, R., i Latorre, A. D., Porte, X. & Fischer, I. Semiconductor laser linewidth reduction by six orders of magnitude via delayed optical feedback. *Opt. Lett.* **42**, 163–166 (2017).
[5] Kelleher, B. & Quinn, G. Mutual coherence enhancement in coupled lasers. *IET Optoelectron.* **11**, 86–90 (2017).
[6] Malakhov, A. N. Influence of the intrinsic noise of oscillators on their mutual synchronization. *Sov. Radiophys.* **8**, 838–846 (1965).
[7] Erzgräber, H., Krauskopf, B. & Lenstra, D. Compound Laser Modes of Mutually Delay-Coupled Lasers. *SIAM J. Appl. Dyn. Syst.* **5**, 30–65 (2006).
[8] D'Huys, O., Jüngling, T. & Kinzel, W. Stochastic switching in delay-coupled oscillators. *Phys. Rev. E* **90**, 032918 (2014).
[9] Henry, C. Theory of the linewidth of semiconductor lasers. *IEEE J. Quantum Electron.* **18**, 259–264 (1982).
[10] Lingnau, B., Ludge, K., Chow, W. W. & Scholl, E. Failure of the alpha factor in describing dynamical instabilities and chaos in quantum-dot lasers. *Phys. Rev. E* **86**, 065201 (2012).
[11] Daly, A. *et al.* Voltage Spectroscopy and the Operating State of an Optically Injected Long Wavelength VCSEL. *IEEE Photon. Technol. Lett.* **24**, 1245–1247 (2012).
[12] Redlich, C. *et al.* Mode-switching induced super-thermal bunching in quantum-dot microlasers. *New J. Phys.* **18**, 063011 (2016).
[13] Albert, F. *et al.* Observing chaos for quantum-dot microlasers with external feedback. *Nat. Commun.* **2**, 366 (2011).
[14] Holzinger, S. *et al.* Tailoring the mode-switching dynamics in quantum-dot micropillar lasers via time-delayed optical feedback. *Opt. Express* **26**, 22457–22470 (2018).
